# Variability in Notification of Positive Newborn Screening Results for Sickle Cell Trait Across the United States

**DOI:** 10.1155/ah/3854629

**Published:** 2024-12-19

**Authors:** Jayla Lynn Scott, Jana Christian, Manuela Plazas Montana, Yvette M. Miller, Rakhi P. Naik

**Affiliations:** ^1^Division of Hematology, Department of Medicine, Johns Hopkins University, Baltimore, Maryland, USA; ^2^Division of Cardiology, Department of Medicine, NYU Langone Health, New York, New York, USA; ^3^Donor and Client Support Center, American Red Cross, Charlotte, North Carolina, USA

**Keywords:** genetic counseling, newborn screening, sickle cell trait

## Abstract

Universal in the United States (US) since 2006, newborn screening (NBS) programs for sickle cell disease (SCD) allow for early identification of the disease and, as an unintentional byproduct, identification of sickle cell trait (SCT). Unlike other carrier states, SCT is highly prevalent and is found in nearly 3 million Americans, which results in important reproductive implications. Currently, all NBS programs in the US are responsible for their own policies regarding SCT notification, and little is known about how SCT notification practices are performed and how these practices vary across NBS programs. We surveyed NBS programs personnel in all 50 states, the District of Columbia, and the US' territories of Puerto Rico and Guam (*n* = 53) using an electronic survey. There was a 100% response rate. All NBS programs (100%) provide notification of SCT status to either a pediatrician or parent: 49% notify the pediatrician only, 45% notify both the pediatrician and parent, and 6% notify the parent only. A total of 98% of NBS programs retain electronic records of SCT status, but only 38% can be directly accessed by pediatricians/primary care doctors. No state operates a publicly available database that allows individuals to access their own records. Only one state provides renotification at reproductive age. In conclusion, there is wide variability in NBS practices for SCT notification. This study demonstrates a need for national guidelines to standardize SCT notification across the US to ensure effective notification and counseling for SCT.

## 1. Introduction

Newborn screening (NBS) programs are used worldwide to identify certain genetic, endocrine, and metabolic disorders that can affect a child's long-term health or survival. NBS programs for sickle cell disease (SCD) have been universal in the United States (US) since 2006 [1, 2]. However, because identification of the carrier state (sickle cell trait (SCT)) is not the primary goal of NBS, there are no guidelines on whether and how to notify individuals and stakeholders about SCT. Among carrier states identified by NBS, SCT is unique as it has high prevalence, has important reproductive implications, and has been found to be a risk factor for select clinical manifestations [3]. Worldwide, an estimated 300 million persons live with SCT, and in the US, an estimated 3 million persons live with SCT. SCT has been found to be associated with chronic kidney disease, venous thromboembolism (VTE), and severe exercise-related injury [4–9]. SCT also has significant reproductive implications as depending on the hemoglobinopathy status of their partner, persons with SCT are at risk of having a child with SCD. Given that SCT becomes relevant again for reproductive planning, many have advocated for renotification of SCT status at reproductive age and/or efficient access to results for SCT status [3].

Little is known about how notification practices for SCT differ between NBS programs. In 2008, two years after the final US state adopted universal screening, Kavanagh et al. published results of surveys of NBS follow-up coordinators in all 50 states and the District of Columbia regarding SCD and SCT notification [10]. They found wide variability in the notification processes between states for SCD and SCT. However, since then, no further studies have reassessed notification processes of US NBS programs for SCT. Renotification practices of NBS programs at reproductive age are also understudied.

It has now been over 15 years since universal NBS for SCD has been established throughout the US, and while our understanding regarding the clinical and reproductive implications of SCT continues to expand, national guidelines for SCT status notification and renotification still do not exist and NBS remains decentralized with each state responsible for its own program [1]. The purpose of our study is to provide an updated understanding of NBS protocols for notification of positive testing for SCT in the US. We hypothesized that there is still significant variability in the notification practices of positive SCT results and that a minority of programs provide renotification at reproductive age or efficient access to SCT results for patients and physicians.

## 2. Methods

We surveyed NBS program personnel in all 50 states, the District of Columbia, and the US territories of Puerto Rico and Guam using an electronic survey developed in Qualtrics. Information for NBS personnel was found using internet research relying mostly on information available on state-specific webpages about NBS and the website Baby's First Test, a US resource center for NBS information. In June 2022, an email sent directly from Qualtrics was sent to the gathered email addresses with a letter describing the study and a personalized link to the survey. Distribution software in Qualtrics revealed that almost half of the emails were undeliverable due to invalid emails. The correct emails for these states were found by calling the NBS program numbers, which had been gathered using internet research, and by coordinating with the manager of the SCD Coalition SCT Task Force, for any contact information they had for NBS professionals in the missing states. A reminder email was sent 2 weeks after initial receipt of the first email for any nonrespondents. Phone calls and email communication were used to gather the remaining nonrespondents from September–November 2022. This study was exempt from review by the Johns Hopkins Medicine Institutional Review Board.

The survey was a mixed methods survey consisting of 47 potential questions with question mapping that enabled respondents to skip questions not relevant to their state (see Appendix 1). The survey was reviewed and refined by a multidisciplinary team. The survey asked questions related to the following: (1) basic demographic information (i.e., title and length of time in their current position), (2) opinions on SCT (i.e., Is SCT a public health concern in their state?), (3) Who is screened for SCD/SCT and how are they screened? (4) Who is informed of a positive SCT result, how they informed, and what is given to them when they are informed? (5) renotification procedures (i.e., Are individuals with SCT renotified later in life, at what age are they renotified?), (6) their NBS program record keeping procedures for SCT statuses (i.e., How is SCT status currently documented and retained?), and (7) funding (i.e., Does their state have dedicated funding for genetic counseling for parents of SCT carriers?).

Responses to the questionnaire were carefully reviewed. For questions in which respondents answered “other,” responses were recoded, using the additional information provided, into existing response categories, if possible. Regarding the question specifically about who is informed of a positive NBS screening result for SCT, the same scheme used by Kavanagh et al. was also employed in our study [10]. In cases where the pediatrician/PCP was notified by the NBS program with the expectation they would notify the parent, the response was coded as “pediatrician/PCP only” for that state. Overall, quantitative data were summarized using descriptive statistics. Qualitative data were reviewed by each author and were categorized into overarching themes, with inclusion of supporting quotes. The protocol for missing data was pairwise deletion to utilize all observed information. Approximately 2.2% (38/1736) of data were missing.

## 3. Results

We received 53 surveys (100% response) from NBS program personnel from all 50 states, the District of Columbia, Guam, and Puerto Rico. Questions about the NBS program for Colorado and Wyoming were completed by the same NBS counselor who represents both states.  Table 1 provides the demographic information for survey respondents including position title and length of time in current position.

### 3.1. Opinion Questions

Most respondents (98%, 52/53) agreed that knowledge of SCT is important for reproductive decision-making, and a majority (66%, 35/53) agreed that SCT status is a public health concern for their state. 92% (49/53) also agreed that more education needs to be done about SCT. However, over 41% (22/53) of respondents disagreed that ensuring counseling about SCT is the responsibility of state NBS programs. The remainder of responses to opinion questions to NBS respondents is further detailed in Table 2.

### 3.2. Testing and Notification Procedures

100% of programs report screening all newborns for SCT with some programs emphasizing that while all newborns are screened, SCT screening is solely a byproduct of SCD screening not the objective. NBS for SCT is mandatory by law for 51% (27/53) of programs, mandatory but can be opted out of in 43% (23/53) of programs, and not mandatory by law in 6% (3/53) of programs. All programs use quantitative or definitive testing to identify hemoglobin variants: 43% (23/53) of programs use isoelectric focusing (IEF), 26% (14/53) use high-performance liquid chromatography (HPLC), 11% (6/53) use both IEF and HPLC, 4% (2/53) use DNA genotyping, and 2% (1/53) use gel electrophoresis. Given the potential for processing or transcription error in NBS results, 47% (25/53) of NBS programs require confirmatory testing either with HPLC or DNA analysis (43%, 23/53) or IEF (4%, 2/53) which is performed either by the NBS program itself or by working with the newborn's physician.

All responding NBS programs (100%) provide notification of SCT status to either a pediatrician or parent, with 49% (26/53) notifying the pediatrician only, 45% (24/53) notifying both the parent and pediatrician, and 6% (3/53) notifying the parent only. Two centers that notify the PCP only also notify their state sickle cell community organization. Parents are most often notified by mail only whereas pediatrician notification most commonly varies from mail only to fax only to phone call in addition to written notification.

Of the NBS programs that notify pediatricians, 42 of the 50 NBS programs provided additional information regarding how notification takes place. The vast majority 88% (37/42) acknowledged that pediatrician information was missing or inaccurate either often (which was described 25%–50% of the time) or sometimes (which was described as < 25% of the time). Reported measures taken by programs to retrieve accurate information include contacting the birth hospital, accessing medical records, or calling parents for information on the current PCP.

**Figure 1:**
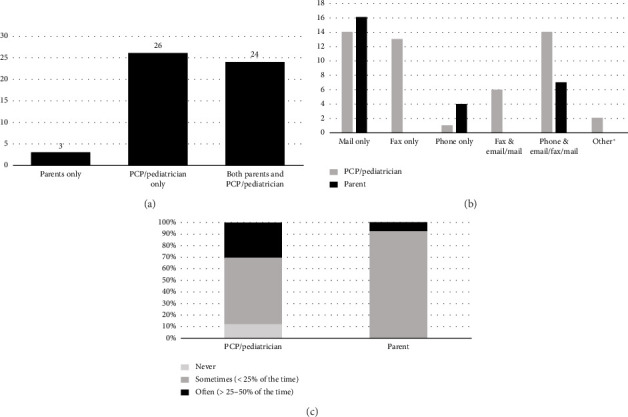
Notification practices of NBS programs for SCT. (a) Who is informed of a positive NBS result for sickle cell trait? (b) How are PCPs and parents informed? ⁣^∗^Secure web portals. (c) How often is contact information missing?.

Of the 27 NBS programs that notify parents, 33% (9/27) provide both written information about SCT and referrals to or resources for local genetic counseling centers, 30% (8/27) provide written/online information about SCT & lab result only, 15% (4/27) provide referral to or resources for local genetic counseling centers only, and 11% (3/27) report providing results only. 90% (26/27) of respondents reported that parent information is missing or inaccurate either often (which was described 25%–50% of the time) or sometimes (which was described as < 25% of the time). Programs reported contacting the birth hospital or using other records like the birth certificate, Medicaid database, or immunization records to retrieve accurate information for parents of babies with positive results. The characterization of how SCT notification occurs is further provided in Figure 1.

Only 2 NBS programs report running their own SCT counseling programs. These two states have state funded sickle cell programs that oversee screening, genetic counseling, and education for SCD, SCT, and other hemoglobinopathies.

### 3.3. Renotification Procedures

Only one state reported routine renotification of SCT carriers at reproductive age. Information about the hemoglobinopathy result and written information about reproductive and/or clinical consequences of SCT is included in renotification. One state reports renotifying the PCP of individuals with SCT when the carrier turns 12 months as mode of ensuring that the PCP who is caring longitudinally for the patient has information on SCT status.

A majority of programs (98%, 49/50) report retaining electronic records of SCT status; however, in over half of programs (55%, 27/49), electronic records are available only after 2000 with the two last states adopting electronic records in 2019. NBS electronic records can be accessed directly by pediatricians/primary care doctors in 38% (18/49) of programs; however, no state operates a publicly available database to allow individuals to access their own records.

### 3.4. Funding and Future Directions

25% (13/53) of NBS programs report having dedicated funding for genetic counseling for parents of SCT carriers. No program reported having funding for renotification of SCT carriers at any stage in life.

When asked what sickle-cell related initiatives they would do if their program had more funding, respondents said they would use the funds for more educational resources and activities, to build databases for both pediatrician and parent electronic access to NBS SCT results, more genetic counseling services, and long-term follow-up initiatives. This is further highlighted in Table 3.

## 4. Discussion

Notification and renotification of SCT results at reproductive age is important to provide carriers with counseling about reproductive choices and to initiate genetic counseling about potential clinical risks. Our results demonstrate significant variability in the process by which NBS programs respond to a positive screen for SCT. Variation between the NBS programs occurs in who is notified, how notification is performed, what information is provided during notification, and whether the positive SCT result has been confirmed by the NBS program. In addition, only one state provides renotification of positive SCT results at reproductive age. This variability in SCT notification and absence of renotification at reproductive age occurs even though the majority of surveyed NBS personnel agree that SCT status is a public health concern for their state (66%), that knowledge of SCT status is important for reproductive decision-making (98%), and that more education needs to be done regarding SCT (92%).

Our findings highlight at least three major barriers to notification of SCT diagnosis. First, inherently, SCT status is a byproduct of NBS programs when testing for SCD, and as a result, less infrastructure exists for the notification of SCT. We saw this reflected in our survey as many of our respondents specifically emphasized that mandatory NBS is for SCD and SCT is a byproduct. Second, the vast majority of NBS programs (94%) aim to notify the primary care physician with or without the parent; however, 32% of the NBS programs responded that the contact information for pediatrician/primary care physician is incomplete or inaccurate 25%–50% of the time, leading to significant hurdles in providing notification. Incomplete and inaccurate contact information also occurs when notifying parents directly. Finally, financial funding is a significant barrier identified with only 25% of NBS programs reporting dedicated funding for genetic counseling for parents of SCT carriers. In addition, no state reports having funding for renotification, which likely limits the ability to renotify persons with SCT at reproductive age.

Our study results expand upon prior work done evaluating the state of NBS programs for SCT. Similar to our study, in Kavanagh's 2008 evaluation of NBS programs, researchers also found significant variability in the notification practices for SCT [10]. This variability in SCT notification found by Kavanagh is replicated in our study and must be addressed given the high prevalence of SCT in the US. In a 2015 study evaluating SCT incidence among newborns over a 20-year period, there were 1,107,875 laboratory reports of possible SCT among the 73,951,175 newborn births screened, which amounted to 1 in 67 [11].

In our survey, 64% of respondents either disagreed or were unsure whether ensuring SCT counseling is the responsibility of NBS programs, and furthermore, only two NBS centers report running their own SCT counseling programs. As such, NBS coordinators/managers heavily rely on pediatricians or community-based organizations to provide important counseling about the significance of SCT. Although there is limited information on how pediatricians/primary care physicians view their role in counseling regarding a positive result of SCT, a 2006 study provides insight regarding the willingness and ability of pediatricians and family physicians to follow up on a positive NBS. In this national mail survey study, many physicians reported that they did not feel comfortable counseling about conditions included in NBS programs, and for SCD specifically, 34.8% of family physicians and 8.8% of pediatricians expressed that they were not competent to discuss the results of a positive NBS result for SCD [12]. Competence in discussing SCT was not elicited in this study. These study results suggest that pediatricians and family physicians may require additional guidance on how to counsel families regarding a diagnosis of SCT. Considering how physicians and NBS programs can work in unison to ensure that SCT counseling occurs will be an important task.

Knowledge of SCT status is accepted to be an important factor for reproductive decision making. In addition, SCT has been found to be a risk factor for select clinical outcomes such as VTE, chronic kidney disease, and rhabdomyolysis [1, 3, 7]. However, our study found that neither renotification at reproductive age nor efficient access to NBS SCT results is available in most states. This raises concern as many prior studies have demonstrated gaps in knowledge among communities regarding personal SCT status [13–16]. A 2016 cross-sectional study of 258 self-identified African American/Black persons of reproductive age found that over half of participants (52%) were uncertain of their personal SCT status and 62% were unsure of the SCT status of their family members [13]. In our analysis, we found that 98% of programs retain electronic records of SCT status but only 38% of programs had records that could be accessed directly by pediatricians/primary care doctors and no program had a database available for individuals to search their own records. The lack of renotification of individuals with SCT may exacerbate knowledge deficits in personal SCT status Furthermore, the lack of physician access to SCT status represents a missed opportunity to provide reproductive and health outcome counseling to SCT carriers.

Limitations of our study include a wide variability in the roles of the NBS personnel who completed the survey, which highlights the lack of standardization in the SCT notification. Our findings were also limited to survey responses, and we did not perform additional interviews which may limit the level of detail and nuance provided in responses. We also limited our investigation to government-run NBS personnel, and in many states, there are nongovernmental organizations that support education and counseling regarding SCT who were not included in this study but who may have provided additional insights into SCT education efforts.

In conclusion, our study demonstrates an urgent need for national guidelines to standardize SCT notification processes across the US to ensure the effective notification and counseling of parents with newborns with SCT. When creating national guidelines for SCT NBS notification, issues which require careful consideration include ensuring sufficient funding and dedicated staff for NBS programs, advancing protocols to confirm that pediatricians and parents receive a positive result, establishing access to important counseling regarding the significance of SCT, instituting a process by which confirmatory testing occurs, and organizing methods to either renotify persons at reproductive age or provide an accessible way to access SCT status at later ages. We hope that by addressing these key issues in improving our NBS SCT notification processes, people with SCT will have access to their important health information paired with genetic counseling on the clinical and reproductive significance of their trait status.

## Figures and Tables

**Table 1:** Characteristics of newborn screening respondents.

Time in current position in years, mean (Q1, Q3)	6.7 (1.9–7.6)
Position title	
NBS program staff, *n* (%)	20 (38)
NBS follow-up program staff, *n* (%)	19 (36)
Nursing staff, *n* (%)	4 (8)
Genetic counselor, *n* (%)	3 (6)
Other,∗*n* (%)	7 (12)

^∗^Position titles in this category are regional coordinator, blood disorders program coordinator, public health program manager, clinical data liaison, technical supervisor, sickle cell program manager, and maternal and child health clinical coordinator.

**Table 2:** Perspectives of newborn screening counselors on sickle cell trait.

	Answer choice	*N* (%)
“SCT is an overall benign condition”	Agree	24 (45)
	Disagree	20 (38)
	Not sure	9 (17)

“SCT is a public health concern for my state”	Agree	35 (66)
	Disagree	9 (17)
	Not sure	9 (17)

“Knowledge of SCT status is important for reproductive decision making”	Agree	52 (98)
	Disagree	0 (0)
	Not sure	1 (2)

“Ensuring counseling about SCT is the responsibility of the state NBS program”	Agree	19 (36)
	Disagree	22 (41)
	Not sure	12 (23)

“I am satisfied with the effectiveness of the NBS SCT notification program for my state”	Strongly agree	9 (17)
	Agree	25 (47)
	Neither agree or disagree	13 (24)
	Disagree	4 (8)
	Strongly disagree	2 (4)

“More education needs to be done about SCT”	Agree	49 (92)
	Disagree	2 (4)
	Not sure	2 (4)

**Table 3:** Responses to “If I had more funding for NBS, how would i use those funds?.”

If NBS programs had more funding, they would…	Exemplar quotes
Invest in more educational resources and activities	“Overall, more education/awareness campaigns, including a specific focus on males and broader reach to other communities of color”

Make SCT results more accessible to PCPs and parents (i.e., via databases)	“We would use the funding to support the requests proposed in pending legislation to create and support a SCT registry. And, possibly, to support the efforts of our state's newborn screening lab in providing individual requests for SCT newborn screening results” “To create a database so that individuals could look up if they have a trait”

Invest in genetic counseling services	“Need a geneticist for our island and genetic counselors too”

Implement long-term follow-up	“Partner with sub-specialist and have a mechanism for maintaining a long-term follow up program for our hemoglobinopathies”

Partner with community organizations	“I would use it to provide a steady contract with a community-based organization to provide the notification and educational efforts these families truly deserve”

## Data Availability

The data that support the findings of this study are available from the corresponding author upon reasonable request.

## Supporting Information

Appendix: Blank version of the survey used in this study which is entitled “SCT Newborn Notification Survey.”
