# Supplementary material for: Variability in Notification of Positive Newborn Screening Results for Sickle Cell Trait Across the United States
Source: Adv Hematol. 2024 Dec 19;2024:3854629. doi: 10.1155/ah/3854629 (PMC11671650; doi:10.1155/ah/3854629)
Supplement: Supporting Information — Additional supporting information can be found online in the Supporting Information section. [file 3854629.f1.docx]

SCT Newborn Notification Survey

Start of Block: Block 1

Q1


 This short survey has been designed to collect information on sickle cell trait notification procedures in newborn screening programs in the United States.

 This survey will take about 10 minutes.  

 We very much appreciate your involvement in this effort.

 **Your completion of this survey or questionnaire will serve as your consent to be in this research study.**

End of Block: Block 1

Start of Block: Default Question Block

Q2 What is your name?

________________________________________________________________

Q3 What is your position title as it relates to newborn screening (NBS)?

________________________________________________________________

Q4 How long have you been in your current position?

________________________________________________________________

Q5 Which states/ territories do you represent? You may select more than one answer.

- Alabama
- Alaska
- Arizona
- Arkansas
- California
- Colorado
- Connecticut
- Delaware
- Florida
- Georgia
- Guam
- Hawaii
- Idaho
- Illinois
- Indiana
- Iowa
- Kansas
- Kentucky
- Louisiana
- Maine
- Maryland
- Massachusetts
- Michigan
- Minnesota
- Mississippi
- Missouri
- Montana
- Nebraska
- Nevada
- New Hampshire
- New Jersey
- New Mexico
- New York
- North Carolina
- North Dakota
- Ohio
- Oklahoma
- Oregon
- Pennsylvania
- Puerto Rico
- Rhode Island
- South Carolina
- South Dakota
- Tennessee
- Texas
- U.S. Virgin Islands
- Utah
- Vermont
- Virginia
- Washington
- West Virginia
- Wisconsin
- Wyoming

Q6 Sickle cell trait is an overall benign condition

- Disagree
- Agree
- Not sure

Q7 Knowledge of sickle cell trait status is important for reproductive decision-making

- Disagree
- Agree
- Not sure

Q8 Sickle cell trait is a public health concern for my state

- Disagree
- Agree
- Not sure

Q9 More education needs to be done about sickle cell trait

- Disagree
- Agree
- Not sure

Q10 Ensuring counseling about sickle cell trait is the responsibility of State NBS programs

- Disagree
- Agree
- Not sure

Q11 I am satisfied with the effectiveness of the NBS sickle cell trait notification program for my state

- Strongly agree
- Agree
- Neither agree nor disagree
- Disagree
- Strongly disagree

| Page Break |  |
| --- | --- |

Q12 Is NBS for sickle cell trait universal in your state - do all newborns get screened for sickle cell trait?

- Yes
- No
- Other: ________________________________________________

Q13 What newborns are screened?

________________________________________________________________

Q14 Is NBS for sickle cell trait mandatory by law in your state?

- No
- Yes
- Yes, but can opt out
- Yes, but must opt in
- Other: ________________________________________________

Q15 Who is informed of a positive NBS screening result for sickle cell trait?

- Parents only
- A specific Pediatrician/PCP only
- Both Parents AND Pediatrician/PCP
- Other: ________________________________________________

Q16 How is the pediatrician/PCP informed?

- Mail
- Email
- Fax
- Phone call
- Other: ________________________________________________

Q17 How often is contact information for the pediatrician/PCP missing or inaccurate?

- Never
- Sometimes (
- Often (>25-50% of the time)
- Very often (50% of the time)

Q18 If contact information is missing, how does your program ensure notification of a physician?

________________________________________________________________

Q19 How is the parent informed?

- Mail
- Email
- Fax
- Phone call
- Other: ________________________________________________

Q20 How often is contact information for the parent missing or inaccurate?

- Never
- Sometimes (
- Often (>25-50% of the time)
- Very often (50% of the time)

Q21 If contact information is missing, how does your program ensure notification of a parent?

________________________________________________________________

Q22 What information is included in the parent notification?

- Hemoglobinopathy result
- Referral to a genetic counseling center
- Website link to information about reproductive and/or clinical consequences of sickle cell trait
- Written information about reproductive and/or clinical consequences of sickle cell trait
- Other: ________________________________________________

Q23 Please list the website link that is provided to parents here.

________________________________________________________________

Q24 Please upload the written information that is shared with parents here.

Q25 How does your state screen for sickle cell trait?

- Hemoglobin Electrophoresis
- High-performance liquid chromatography
- DNA analysis
- Other: ________________________________________________

Q26 In your state, does a newborn screening result indicating sickle cell trait require confirmatory testing with a more quantitative method such as hemoglobin electrophoresis, high-performance liquid chromatography or DNA analysis?

- Yes
- No
- Other: ________________________________________________

Q27 Does your NBS program ensure that confirmatory testing is performed?

- Yes
- No, the responsibility lies with the PCP/pediatrician and/or parent.
- No, other: ________________________________________________

Q28 In your state, is a baby’s sickle cell trait status routinely entered into their electronic medical record or the NBS state database?

- Yes
- No
- Other: ________________________________________________

Q29 Does your NBS program have dedicated funding for genetic counseling for parents of sickle cell trait carriers?

- Yes
- No

Q30 Who/what provides this funding?

- State/government
- Private
- Other: ________________________________________________

| Page Break |  |
| --- | --- |

Q31 Are individuals with sickle cell trait re-notified later in life?

- Yes
- No
- Other:

Q32 At what age do you renotify?

________________________________________________________________

Q33 How is the sickle cell trait carrier re-notified?

- Mail
- Email
- Fax
- Phone call
- Other:

Q34 How often is contact information for the re-notification missing or inaccurate?

- Never
- Sometimes (
- Often (>25% of the time)
- Very often (50% of the time)

Q35 What information is included in the re-notification?

- Hemoglobinopathy result
- Referral to a genetic counseling center
- Website link to information about reproductive and/or clinical consequences of sickle cell trait
- Written information about reproductive and/or clinical consequences of sickle cell trait
- Other: ________________________________________________

Q36 Please list the website link that is provided to individuals here.

________________________________________________________________

Q37 Please upload the written information that is shared with individuals here.

Q38 Does your NBS program have dedicated funding for renotification of sickle cell trait carriers?

- Yes
- No

Q39 Who/what provides this funding?

- State/Government
- Private
- Other: ________________________________________________

| Page Break |  |
| --- | --- |

Q40 Is sickle cell trait status documented and retained in a records database?

- Yes
- No
- Other: ________________________________________________

Q41 How is sickle cell trait status currently documented and retained?

- Paper
- Electronic
- Paper & Electronic
- Other: ________________________________________________

Q42 From which year forward is sickle cell trait status available electronically? (I.e. 1980, 1981, etc)

________________________________________________________________

Q43 Who can access this electronic record? 

- NBS program personnel only
- NBS program personnel and parents
- NBS program personnel and PCPs
- NBS program personnel, parents, and PCPs
- Other: ________________________________________________

Q44 In your state, is there a publicly available interface where individuals can look up their sickle cell trait status?

- Yes
- No
- Other: ________________________________________________

Q45 If you had more funding for sickle cell trait-related initiatives through your NBS program, what would you use it for?

________________________________________________________________

Q46 Are there any other sickle cell trait-related initiatives that your state provides that we did not previously ask about? Or are there any additional thoughts you would like to provide?

________________________________________________________________

Q47 We are interested in interviewing select NBS programs using a semi-structured phone interview to gather additional data. Would you be interested in us contacting you for this purpose? (Saying yes is a nonbinding commitment)

- Yes
- No

End of Block: Default Question Block
